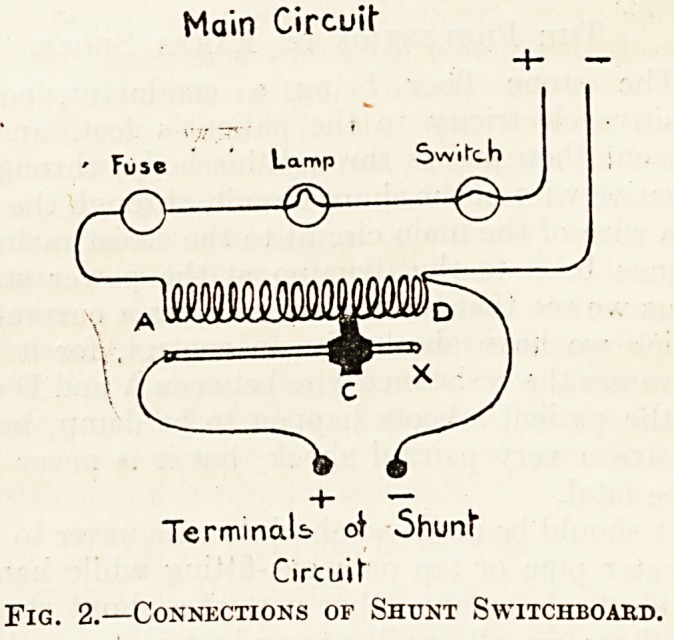# Continuous Current from the Main

**Published:** 1912-03-09

**Authors:** Alfred C. Norman

**Affiliations:** House Surgeon at Sunderland and Durham County Eye Infirmary, Sunderland.


### ELECTRICITY IN MODERN MEDICINE.*

### VIII.—Continuous Current from the Main.

By ALFRED C. NORMAN, M.D. Edin., House Surgeon at Sunderland and Durham County Eye Infirmary, Sunderland.

Fig. 1 illustrates a simple and portable type of shunt switchboard for using continuous current from the main for all forms of galvanisation, ionic medication, and electrolysis. It consists of a board about 6 by 10 inches, on which are mounted a shunt rheostat and a resistance lamp, as well as a switch, a safety fuse, and a pair of terminals for connecting the patient with the shunt circuit. The switchboard can be connected to any electric lamp-holder in a house or hospital by means of a plug adaptor and some flexible wire, and it consumes less than ¼ ampère of current.

Fig. 2 is a diagram of the connections of this switchboard, which, if taken in conjunction with fig. 2, p. 510, of The Hospital of February 17 hardly requires further explanation. The rheostat consists of several hundred turns of a very fine German-silver wire coiled round a flat slate core, A-D. The movable contact C, which slides along the fixed brass rod X, collects current from some part of the wire between A and D and transmits it to the brass rod X, whence it is conveyed by a wire to one of the terminals of the shunt circuit. An incandescent carbon lamp is placed in the main

Fig. 1.—Shunt Switchboard for Galvanisation.

circuit of the rheostat to act as an extra resistance. This is necessary to protect the wire of the rheostat, which is so fine that it would be overheated if it were connected directly to the 220-volt electric-lighting supply. A 16-c.p. lamp for a 220-volt supply has a resistance of, roughly, 800 ohms, and this is the lamp we should use for our present purpose. The resistance of the fine wire in the rheostat is about 400 ohms, so we have a total resistance in the main circuit of about 1,200 ohms, and this allows a current of less than ¼ ampère (180 milliampères, to be exact) to traverse the main circuit of the rheostat; but this small ampèrage is quite sufficient for our purpose, since we practically never require for galvanisation, etc., a current as great as 100 milliampères in the shunt circuit. When the sliding contact is at the end of the wire, D, the voltage between the terminals of the shunt circuit will be 0. As the contact is moved towards the other end of the wire the voltage in the shunt circuit will gradually increase until at the extreme end, A, it will be about 73 volts; that is to say, one-third of the original 220 volts, since one-third of the total resistance in the main circuit lies in the resistance wire between A and D, the remaining two-thirds being in the lamp.

By using many turns of a very fine wire in the rheostat the drop in pressure between one turn and the next is less than one-tenth of a volt; hence, by moving the contact only the distance of a turn or so at a time, the voltage in the shunt circuit can be raised by such very gradual increments that the patient feels no shock, and the number of milliampères which he receives is absolutely under control.

A shunt rheostat should always be used in conjunction with a milliampèremeter to indicate how much current the patient is receiving. The meter may be a fixture on the board (in the shunt circuit, of course), or it may be joined up in series with the

Fig. 2.—Connections of Shunt Switchboard.

patient when required. When a shunt rheostat is to be used for testing the electrical reactions of muscles it should also be fitted with a current reverser, so that the active electrode may be quickly changed from positive to negative without unfastening the connections.

A switchboard similar to fig. 1 costs about £2 15s. A larger switchboard with milliampèremeter and current reverser permanently mounted in the shunt circuit would cost about £6 10s.

For use in a private consulting-room where there is usually a carpet on the floor, or in the wards of a hospital with wood floors, these shunt rheostats are very satisfactory, but in rooms with stone, tiled, or terrazzo floors they have to be used with certain precautions, because these floors are good conductors of electricity, and it is possible to give a patient a very uncomfortable " earth " shock if he be not properly insulated. This may happen in the following way:—

In most houses supplied with electric light there is some leakage of current to earth, and in some

* Previous articles in this series have appeared in The Hospital of Nov. 11 and 25, Dec. 9 and 30, Jan. 13 and 27, and Feb. 17.

power stations they purposely connect one pole of the dynamo to earth for certain purposes. We have seen that current generated at one pole of a dynamo or battery always tends to find its way to the other pole by the path of least resistance, and we know that the earth is an excellent conductor of electricity, so that current liberated from a wire connected with, say, the positive pole of a dynamo will travel for miles through the earth to find its way back to the negative pole. Let us suppose that there is a leak of electricity from a positive wire in some house or power station in a certain town, and that a patient standing on a stone floor is to be treated with current from a shunt rheostat connected with the mains as shown in fig. 2. The earth will be charged with a positive current, whose object is to get back by the shortest route to the negative pole of its generator—*i.e.* the dynamo at the power station; and as soon as we touch the patient with an electrode connected with the negative terminal of the shunt circuit this current will pass through his body, even when the sliding contact is at D. The diagram (fig. 2) shows how this may happen.

### The Prevention of Earth Shock.

The stone floor being a conductor, conveys positive electricity to the patient's feet, and this current then passes through his body, through the negative wire of the shunt circuit, through the negative wire of the main circuit to the street main, and thence back to the dynamo at the power station. Thus we see that the patient receives a current over which we have absolutely no control, for it never traverses the resistance wire between A and D at all. If the patient's boots happen to be damp, he may receive a very painful shock, but it is never likely to be fatal.

It should be made an absolute rule never to touch a water pipe or tap or a gas-fitting while handling an electrode or any other part of a shunt rheostat, for these are all excellent conductors to earth, and could give rise to severe " earth " shocks in the manner described above.

Current from a shunt rheostat should never be used to give an electric bath except by an expert who realises and knows how to guard against the dangers of an " earth " shock. With a patient immersed in water such a shock might be very severe, or even fatal.

For ordinary galvanic treatment a patient can be perfectly insulated by making him stand on a cork mat. Another way to safeguard the patient is to connect the switchboard to the mains in such a way that the " weak " end of the rheostat (D in the diagram) is of the same polarity as the earth current. This is quite a simple matter. The surgeon, while standing on a stone floor, should move the sliding contact to " weak " and touch each terminal in turn with a moistened finger. If he gets a shock the plug adaptor connecting the switchboard to the main supply should be reversed. But in small hospitals where electrical apparatus may have to be used by people who, to say the least, are not expert, it is safer to instal a motor generator, such as the Pantostat, because it is impossible to give a patient an earth shock with this type of apparatus.

A galvano cautery may be worked by means of a specially constructed shunt rheostat, but this is a clumsy and wasteful piece of apparatus. To obtain 18 ampères or so of sufficiently low-voltage current in the shunt circuit we should have to pass more than 50 ampères through the main circuit, and this, apart from the waste of current, would require an expensive installation of especially heavy house cables.

By far the most satisfactory method of adapting the continuous current for cautery purposes is to use a motor transformer of the Pantostat type.

Medical lamps for diagnosis can be very satisfactorily lighted by current from a shunt switchboard similar to the one shown in fig. 1, but since more current is required for medical lamps than for galvanisation, the rheostat is constructed of stouter wire, and a 60 candle-power lamp is placed in the main circuit. But here again there is a risk of " earth " shocks if we touch the metal parts of a cystoscope, head lamp, or hand lamp connected with a shunt rheostat. By standing on a cork mat, we can of course obviate this risk, but still it is safer in hospital to use current from a motor generator.

### The Pantostat.

This type of motor generator consists of a motor and a dynamo wound side by side on one axle, but completely insulated from each other. Current from the main is used to drive the motor, and the motor revolves the armature of the dynamo. The dynamo generates an entirely new current of suitable ampèrage and voltage for practically all electro-medical requirements. The great advantage of this type of apparatus lies in the fact that there is no chance of giving a patient an " earth " shock, because electricity generated at one pole of a dynamo tends to find its way back to the opposite pole of that dynamo, *and no other*. Hence an earth current produced by leakage from a dynamo at the power station would not pass from earth through a patient's body in order to reach the dynamo of a motor generator. Nor would current generated in the latter pass to earth through the patient's body so as to reach the dynamo at the power station.

The Pantostat is mounted on a cast-iron base, on which there are four pairs of terminals, labelled: " Galvanisation," " Faradisation," " Light," and " Cautery " respectively. Mounted in the base are five sliding rheostats. The first is used to control the speed of the motor; the other four are for regulating the current at the various terminals. Thus at the cautery terminals we can obtain a current up to 30 ampères at less than 10 volts; at the light terminals we can obtain any current up to 2 ampères at any voltage up to 20; at the terminals for galvanisation we can get a current varying from one-tenth of a m.a. to 100 m.a., and there is also a milliampèremeter and a current reverser in this circuit, so that the apparatus can be used for delicate muscle-testing as well as for the most powerful applications used in ionic medication.

*(To be continued.)*

## Figures and Tables

**Fig. 1. f1:**
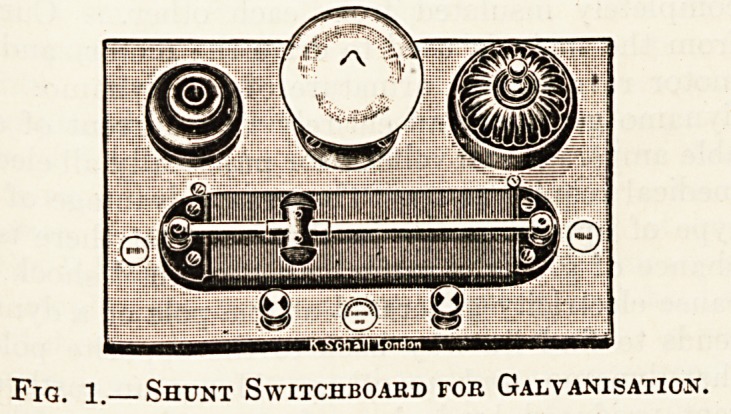


**Fig. 2. f2:**